# Solid-basaloid variant of adenoid cystic carcinoma of the breast with near complete response to neoadjuvant chemotherapy

**DOI:** 10.1038/s41523-022-00469-z

**Published:** 2022-08-11

**Authors:** Anne Grabenstetter, Edi Brogi, Hong Zhang, Pedram Razavi, Jorge S. Reis-Filho, Kimberly J. VanZee, Larry Norton, Hannah Y. Wen

**Affiliations:** 1 Department of Pathology and Laboratory Medicine, Memorial Sloan Kettering Cancer Center, New York, NY 10065 USA; 2 Department of Medicine, Memorial Sloan Kettering Cancer Center, New York, NY 10065 USA; 3 Department of Surgery, Memorial Sloan Kettering Cancer Center, New York, NY 10065 USA

**Keywords:** Breast cancer, Surgical oncology

## Abstract

Breast adenoid cystic carcinoma (AdCC) is a rare subtype of triple negative breast cancer. Two morphologic variants are described, namely classic AdCC (C-AdCC) and solid basaloid (SB-AdCC). Recent studies have shown that the SB-AdCC variant has significantly worse prognosis than C-AdCC. Due to the rarity of SB-AdCC, no standard recommendations are available for its management. Data on the use and benefit of chemotherapy in patients with SB-AdCC are sparse and the response to neoadjuvant chemotherapy has not been reported. We present the clinical and pathologic findings of a patient with SB-AdCC treated with neoadjuvant chemotherapy who achieved a remarkable pathologic response.

## Introduction

Breast adenoid cystic carcinoma (AdCC) is a rare histologic subtype of breast cancer, usually of triple negative phenotype. These tumors account for <1% of all breast cancers, composed of a dual population of epithelial and myoepithelial neoplastic cells, arranged in cribriform, tubular and/or solid growth patterns. AdCCs have been shown to be driven by activation of the MYB pathway, most commonly due to a *MYB-NFIB* fusion gene, followed by fusion genes involving *MYBL1* or *MYB* gene amplification^1^. The solid basaloid variant of AdCC (SB-AdCC) is composed of cells with scant cytoplasm, marked nuclear atypia and increased mitotic activity^2^. In contrast to the classic form of AdCC (C-AdCC), which typically follows a relatively indolent course, the SB-AdCC variant has an aggressive clinical behavior^2–4^. Vanishingly rare (there are less than 100 published cases of SB-AdCC), currently there are no standardized recommendations regarding management. In the limited series available, most patients were treated with surgical excision and post-operative radiation but adjuvant chemotherapy was often omitted, similar to the management of C-AdCC^5–7^. At present, there is a paucity of data on chemotherapy benefit for SB-AdCC and its response to neoadjuvant treatment is unknown. Here, we describe a case of SB-AdCC treated with neoadjuvant chemotherapy with a near complete pathologic response.

## Results

### Case presentation

A 56 years old woman of Ashkenazi Jewish descent with no significant past medical history or family history of breast or ovarian cancer, initially presented to an outside hospital after palpating a right breast mass. The patient underwent bilateral mammogram that revealed a right breast 9 mm spiculated mass with associated calcifications and nipple retraction, corresponding to the sonographic finding of an 8 mm irregular, retroareolar mass which appeared to invade the nipple-areolar complex. Ultrasound of the right axilla showed a suspicious lymph node. An ultrasound guided biopsy of the breast mass yielded an infiltrating moderately differentiated mammary carcinoma with associated intermediate grade intraductal carcinoma, solid and cribriform type. Subsequently, the patient presented to Memorial Sloan Kettering Cancer Center (MSK) for cancer care.

**Fig. 1:**
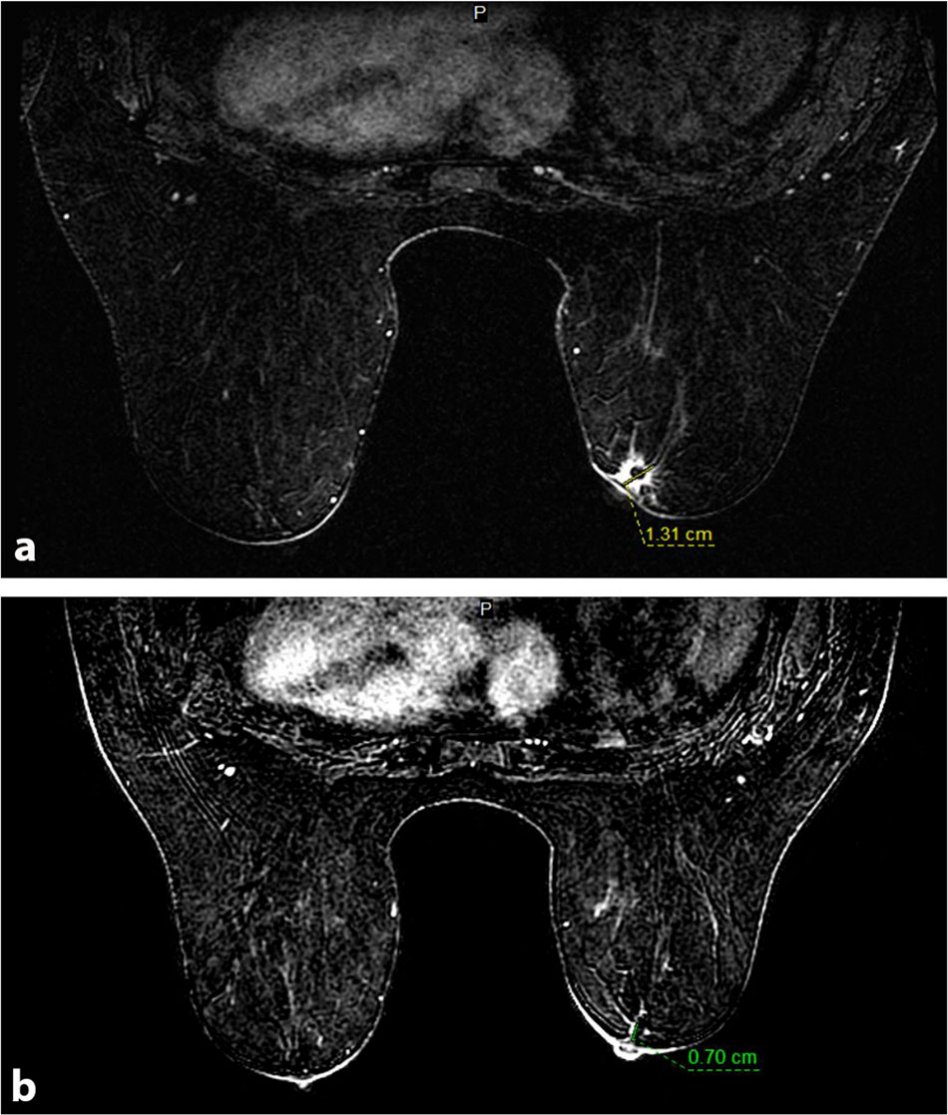
Pre and post neoadjuvant chemotherapy imaging. **a** Pre-therapy MRI showing retroareolar, irregular enhancing mass, 1.3 cm; **b** Post-therapy MRI showing faint linear enhancement, 0.7 cm.

**Fig. 2:**
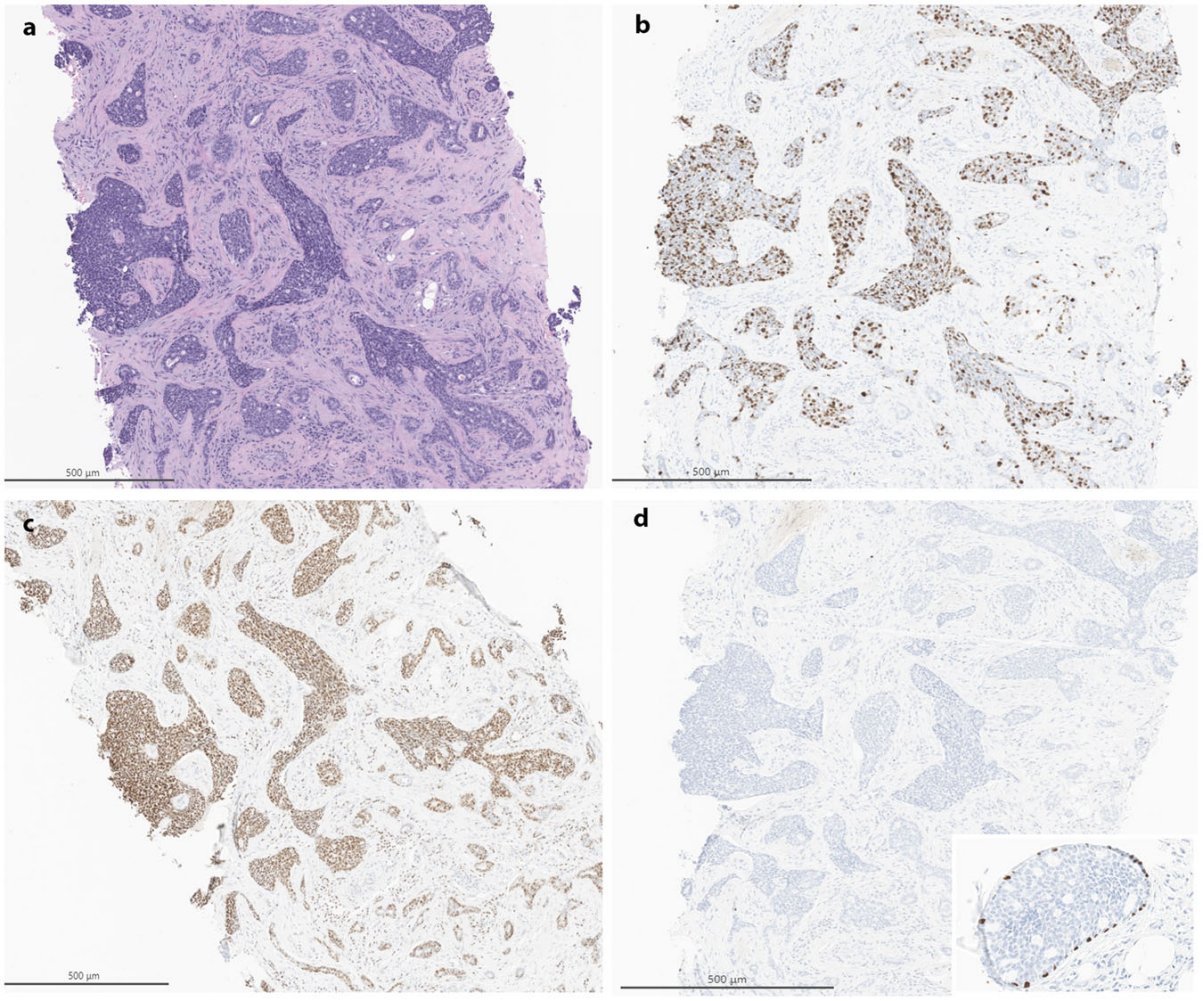
Core needle biopsy, treatment naïve. **a** Core biopsy showing infiltrating basaloid carcinoma with solid and cribriform nests (H&E, 50X); **b** Ki67 proliferation index of approximately 40% (50X); **c** Diffuse nuclear expression with MYB immunohistochemistry (50X); **d** p63 immunohistochemistry demonstrates loss of myoepithelial cells surrounding invasive nests, inset reveals focal in situ carcinoma with rim of p63 positive myoepithelial cells (50X, inset 200X).

Physical examination revealed a palpable right breast mass measuring approximately 10 mm immediately deep to the nipple, focally inseparable from the skin. There was no obvious palpable adenopathy. Breast MRI revealed a 13 × 13 × 12 mm irregular enhancing mass with T2 hyperintensity in the retroareolar region along with an abnormal right level 1 axillary lymph node, measuring 10 mm (Fig. 1). As per protocol, the initial core biopsy was reviewed by the MSK Pathology Department. Microscopically, multiple tissue cores were involved by an infiltrating basaloid carcinoma with solid nests and focal cribriform architecture within a desmoplastic stroma showing approximately 5–10% stromal tumor infiltrating lymphocytes (Fig. 2). The neoplastic cells displayed a high nuclear to cytoplasmic ratio, moderate nuclear atypia and conspicuous mitoses. Immunohistochemistry (IHC) demonstrated diffuse expression of cytokeratin 7 (CK7), CK8/18, CK5/6 and c-kit (CD117), occasional p63 reactivity, and MYB nuclear expression (Fig. 2). A *MYB* translocation was identified by fluorescence in situ hybridization (FISH) utilizing break apart probes. The tumor lacked estrogen receptor (ER), progesterone receptor (PR) and human epidermal growth factor receptor-2 (HER2) expression. The Ki67 proliferation index was approximately 40% (Fig. 2). The histologic, IHC and molecular findings supported a diagnosis of SB-AdCC. Focal in situ carcinoma was also identified. Fine needle aspiration (FNA) of the suspicious right axillary node yielded malignant cells, consistent with metastatic carcinoma.

Breast conservation surgery (BCS) and mastectomy, along with sentinel lymph node biopsy were discussed with the patient. Due to the central location of the tumor and imaging suggesting involvement of areolar skin, a wide excision would also require removal of the nipple areolar complex. In this context, a medical oncology consultation was obtained. Given the triple negative phenotype and biopsy proven positive lymph node status, neoadjuvant chemotherapy was recommended because if it could provide a complete pathologic response in the axilla that would obviate the need for axillary dissection. The patient elected to have an attempt at BCS and thus was given pre-operative dose-dense AC-T (doxorubicin plus cyclophosphamide each two weeks with G-CSF support for four cycles followed by carboplatin each 3 weeks for four cycles plus twelve cycles of weekly paclitaxel), which was well tolerated. Because AdCC is often regarded to be a chemotherapy-resistant subtype of breast cancer the patient was monitored with monthly imaging studies to assess response and ensure there was continued shrinkage of disease. After FDA approval, she was also started on pembrolizumab. However, she developed immune-mediated nephritis after the second dose of pembrolizumab resulting in its discontinuation. An MRI was performed following completion of therapy which showed very faint, residual 7 mm linear enhancement extending towards the nipple, significantly decreased from prior study (Fig. 1). On pre-surgical physical examination, the palpable mass was no longer appreciated, the areolar skin did not feel tethered to the underlying breast tissue and there was no axillary adenopathy. The patient underwent breast conserving surgery with removal of nipple and sentinel lymph node biopsy upon completion of neoadjuvant treatment.

**Fig. 3:**
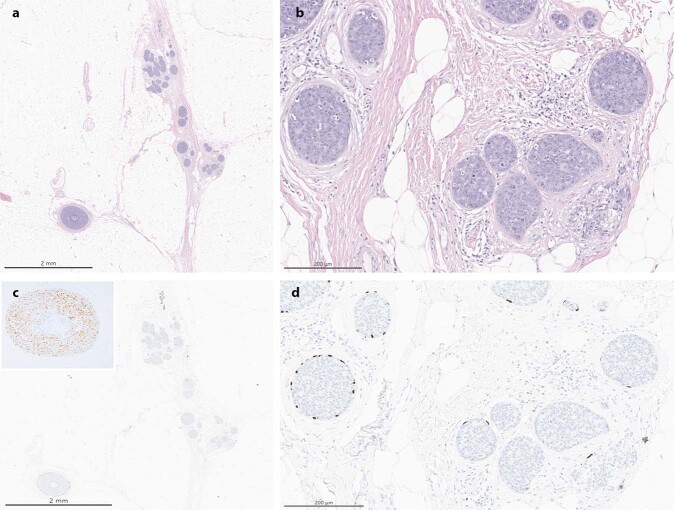
Excision specimen, post neoadjuvant chemotherapy. **a** Residual carcinoma, H&E, 10X; **b** Residual invasive (lower right corner) and in situ carcinoma, H&E, 100X; **c** p63 immunohistochemistry, 10X; inset MYB in situ hybridization, 100X; **d** p63 immunohistochemistry, 100X.

The resection specimen obtained post-neoadjuvant treatment showed a single focus of residual invasive carcinoma, spanning <0.5 mm microscopically, along with approximately 5 mm of residual in situ carcinoma, present in a fibrotic tumor bed grossly measuring 12 × 7 mm (Fig. 3). The carcinoma harbored a MYB rearrangement as detected by in situ hybridization and lacked ER, PR and HER2 expression by IHC. Lymphovascular invasion was not identified. The margins of resection were uninvolved by carcinoma. Three sentinel lymph nodes were retrieved and showed no residual metastatic disease. Treatment related changes were not identified in the lymph nodes. The patient was referred to radiation oncology and received adjuvant radiation therapy to 50 Gray in 25 fractions. The patient developed mild erythema during radiotherapy but otherwise tolerated it well. She is currently six months status post operation and has recovered well from neoadjuvant chemotherapy and radiation without major complications. She was then started on adjuvant capecitabine, which she is tolerating well. She has remained clinically disease free and is undergoing active surveillance as per guidelines.

## Discussion

The mammary SB-AdCC variant was first described in a report by Shin and Rosen^2^, in which they detailed 9 cases collected over a period of 11 years. While none of the patients in their series had evidence of local recurrence or distant metastases despite a follow up period of up to 7.3 years (mean 32 months), the data presented in that study suggested that SB-AdCC had a higher propensity for lymph node metastases than C-AdCC. Subsequent studies confirmed this observation, which lead to the recommendation for routine axillary staging in these patients, and also noted more aggressive clinical behavior^3,6,8^. In the largest series reported to date, Schwartz et al.^6^ compared the clinicopathologic features and follow-up data of 29 SB-AdCC and 75 C-AdCC from MSK. SB-AdCCs had significantly higher histologic grade, higher rates of lymphovascular invasion and lymph node metastases, and were more likely to recur locally and develop distant metastatic disease. In contrast, C-AdCC had a favorable clinical course, with higher survival rates than invasive breast carcinoma of no special type, and 5- and 10-year disease free survival (DFS) rates greater than 95 and 90%, respectively^1,8^. In the cohort described by ref. ^6^, the surgical approach and use of adjuvant radiotherapy were similar between the variants, however patients with SB-AdCC were significantly more likely to receive adjuvant chemotherapy than C-AdCC (68.4% versus 17.3%, respectively). Despite this, DFS was significantly reduced in patients with SB-AdCC, with a median DFS of 46.5 months for SB-AdCC versus 151.8 months in those with C-AdCC. While the optimal chemotherapy regimen and benefit of chemotherapy for SB-AdCC are unknown, the poor outcomes of the solid and basaloid variant suggest systemic therapy may be warranted. We present a case of SB-AdCC treated with platinum and anthracycline containing neoadjuvant chemotherapy which achieved an excellent pathologic response, showing complete resolution of the patient’s axillary disease and less than 0.5 mm of residual invasive carcinoma.

Published data on the effect of chemotherapy in patients with SB-AdCC is based on small trials and more frequently involve AdCC arising in organs other than the breast. Laurie et al.^9^ performed a systematic review of 34 studies, including prospective and retrospective data, examining the systemic treatment of metastatic or locally recurrent AdCC, all but one of which included only tumors of salivary gland origin. In the trials which looked at single agent cytotoxic therapy, the observation of stable disease, noted in 58% (64/111) of patients, was more common than objective major responses which were seen in only 13% (18/141) of patients. Overall, in trials utilizing combination therapy, 25% of patients responded, most commonly to regimens composed of cisplatin, doxorubicin and cyclophosphamide (CAP)^9^. These results should be interpreted with caution, though, as none of these multi-agent trials enrolled more than 12 patients. The authors also found discrepant response rates amongst reports, with higher rates observed in retrospective reviews versus clinical trials and ultimately conclude further study is needed to develop a standardized management approach. The patient reported here had a remarkable response, providing further support to the notion that chemotherapy may be effective in at least a subset of SB-AdCCs.

Additionally, novel drug therapies targeting specific molecular alterations may prove to be efficacious in the rare SB-AdCC. The translocation t(6;9)(q23;p23) leading to the *MYB*-*NFIB* fusion gene is identified in up to 86% of C-AdCC and has been confirmed to be present in the solid-basaloid variant as well, however it is seen much less frequently, reported in only 12.5% of cases in one series^10^. Additionally, MYB expression by IHC has been observed in up to 63% of other basal-like, triple negative breast cancers and is not pathognomonic for SB-AdCC^11^. We do not recommend using FISH or IHC testing for MYB unless there are morphologic features strongly raising the differential for SB-AdCC, to avoid overdiagnosis. The most definitive feature pointing to the diagnosis is the identification of areas showing C-AdCC features, particularly identifying the dual cell population and pseudolumina filled with basement membrane material. In the core biopsy presented here the tumor showed focal “cribriforming” areas along with typical basaloid cytologic features suggesting the diagnosis. Another feature of SB-AdCC is the characteristic nested growth pattern embedded in a markedly desmoplastic stroma which contrasts with the typically solid, dense mass seen in poorly differentiated triple negative tumors of no special type.

While there are currently no drugs available to target the *MYB* gene, recent analysis has revealed that *ATR* gene expression is significantly upregulated in *MYB* overexpressing cells^12^. Andersson et al.^12^ observed a dose dependent decrease in proliferation and increase in apoptosis following inhibition of *ATR*, a gene involved in DNA damage repair, and in vivo experimentation demonstrated significant tumor suppression and, in one case, tumor regression, supporting the development of therapeutic targets to *ATR* in the treatment of C-AdCC and possibly a subset of SB-AdCC. Furthermore, it has been reported that the mutation rate of *NOTCH* genes is significantly higher in SB-AdCC compared to C-AdCC^3^. Clinical trials involving Notch signaling inhibitors are currently ongoing and may show usefulness in this variant^3^.

In conclusion, SB-AdCC is a rare subtype of AdCC. While scant data on optimal management are available, we demonstrate that neoadjuvant chemotherapy may induce an excellent response. Our case suggests that combination chemotherapy has a role in treatment. If ongoing studies are successful, additional targeted therapies could provide desirable outcomes in this more aggressive subtype.

## Methods

### Data collection

Written informed consent was obtained from the patient to publish this case report and associated clinical details. Clinical and radiologic data for this case were obtained from the electronic medical record. IHC was performed according to manufacturer’s manual. FISH was done using DNA breakapart signal analysis.

## Data Availability

All the data supporting the findings in this case report are contained within the text.
